# Wound Myiasis in Severe Venous Stasis Ulcer

**DOI:** 10.7759/cureus.8585

**Published:** 2020-06-12

**Authors:** Jennifer C Asotibe, Ikechukwu Achebe, Chimezie Mbachi, Rita Igwilo, Isaac Paintsil

**Affiliations:** 1 Medicine, John H. Stroger, Jr. Hospital of Cook County, Chicago, USA; 2 Internal Medicine, John H. Stroger, Jr. Hospital of Cook County, Chicago, USA; 3 Internal Medicine, University of Nigeria, Enugu, NGA

**Keywords:** myiasis, venous stasis ulcer, venous insufficiency, maggots, infestation, larval debridement therapy, maggot debridement therapy

## Abstract

Although myiasis infestation of wounds presents with significant psychological discomfort to patients, studies have shown that it can be beneficial in the management of recalcitrant ulcers resistant to standard management. Here we report a patient with persistent ulcers unresponsive to standard management who was lost to follow-up for five months and presented with ''maggots in his wound''. This however proved beneficial to the patient as the ulcer showed healthy granulation tissue on presentation and improved healing on follow-up. Our case presents the beneficial effect of myiasis infestation in the 21st century and helps to highlight a time-tested therapy with further encouragement of the use of biotherapy (sterile maggots) for the management of recalcitrant ulcers.

## Introduction

Venous stasis ulcer is the common and costly complication of chronic venous insufficiency. As stasis ulcers account for nearly 70% of chronic lower extremity ulcerations, increasing emphasis is being placed on investigations and innovation of new strategies to better manage this disease [1]. This report details a rare presentation of a patient with chronic, bilateral, medial malleolar venous stasis ulcers, who had undergone multiple debridements and great saphenous vein stripping with little to no improvement of his ulcers. After months of missed appointments and poor wound care, the patient returned to the hospital with maggot infestation of his left medial malleolar venous ulcer.

## Case presentation

The patient described is a 69-year-old male with hypertension, diabetes mellitus type II, and severe chronic venous stasis, complicated by ulceration, who presented with severe pain around his left lower extremity ulcer. The pain was chronic, intermittent, and had worsened over the past week. On examination, we noticed an uncountable number of maggots in his left leg ulcer. He had no fever, chills, swelling, erythema, or wound drainage from the ulcer site. 

This patient had multiple admissions for the treatment of bilateral medial malleolar venous stasis ulcers. His last admission was approximately one year prior, during which he had undergone extensive debridement of bilateral non-healing lower limb ulcers, in addition to right great saphenous vein stripping from the saphenofemoral junction. He was eventually discharged with plans for regular follow-up at the vascular clinic. Over the five months following his procedure, the patient had poor wound healing and was lost to follow-up.

On examination, vitals were normal. Generally, the patient was well appearing. Lower extremities were edematous with prominent varicose veins and stasis dermatitis. His lower extremities revealed chronic ulcerations at the level of the medial malleolus bilaterally. The left lower extremity had a prominent 14 cm x 7.5 cm ulcer above the medial malleolus, with live maggots residing in its crater. The ulcer floor, however, revealed healthy pink granulation tissue without drainage or swelling. 

**Figure 1:**
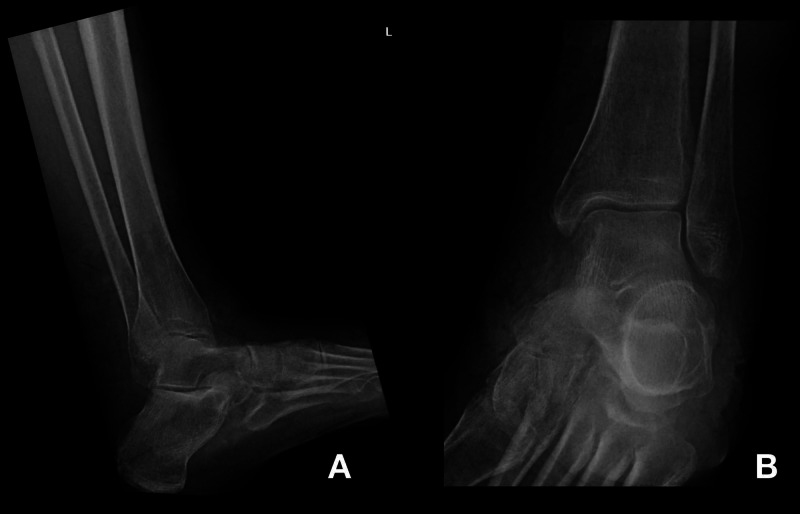
Plain radiograph of lateral (A) view and medial (B) view of the left ankle. No acute fracture or dislocation. Diffuse soft tissue swelling and subtle cortical erosions involving the medial aspect of the navicular bone as may be seen in osteomyelitis.

Initial X-rays of the left ankle revealed severe soft tissue swelling with subtle cortical erosions involving the medial aspect of the navicular bone suspicious for osteomyelitis (Figure 1).

Pertinent laboratory values were as follows: white blood cell (WBC) = 8.6 K/uL, hemoglobin (Hb) = 8.0 g/dL, platelets = 230 K/uL, creatinine = 0.8 mg/dL, blood urea nitrogen (BUN) = 35 mg/dL (H). Blood cultures were taken, and the patient was empirically started on vancomycin in the emergency department (ED). 

After removal of the maggots from the ulcer, by the surgical team, dramatic improvements in wound healing were found and the previously noted necrotic wound bed was replaced with healthy granulation tissue. 

After debridement, the ulcer was dressed and wrapped. The patient’s hospital course continued without fever or leukocytosis. Antibiotics were stopped after cultures remained without growth. The patient was eventually discharged with follow-up, and instruction for wound care. 

During follow-up in the vascular clinic a week later, the patients ulcer had reduced further in size (13 cm x 7 cm x 0.2 cm), and continued to show further signs of healing.

## Discussion

Myiasis is a parasitic infection of body tissues by dipterous larvae (maggots). The fly species most typically found in the human host include Dermatobia hominis (human botfly) and Cordylobia anthropophaga (tumbu fly) species [2]. Infestation typically occurs through accidental deposition of egg larvea into necrotic tissue, open wounds, and even intact skin. These environments, in combination with tropical humid weather, provide a comfortable niche for larvae growth and prosperity [3,4]. Medical conditions predisposing to myiasis include diabetes, hypertension, peripheral arterial disease, and chronic venous insufficiency. Through a cascade of microcirculatory dysfunction, inflammation, and necrosis, conditions are set for the formation of ulcers that are notoriously difficult to manage [1].

Ulcer treatment is typically twofold. First, heal the wound. Second, prevent recurrence. This is accomplished using a combination of rigorous wound care, antibiotics, debridement, and grafting. Not only are these procedures costly, but with approximately 30% of healed ulcers recurring in the first year, and 78% within two years, the efficacy of these strategies is variable [1]. This case highlights maggot therapy as an alternate approach to venous ulcer management that is currently underutilized clinically. 

Maggot use in wound care dates as far back as the 14th century when they were used by the Mayans of Central America and Aboriginal tribes from Australia [5]. William Baer, an orthopedic surgeon during World War I, was the first clinician to use maggots systematically after witnessing how soldiers with maggot-infested wounds fared better than those without. Presentation of his work popularized the technique and led to over 300 American hospitals introducing maggots into their wound healing programs by 1940 [6-8].

Maggots facilitate healing through debridement, disinfection, and stimulation of vascular regrowth [3]. As they consume necrotic tissue, they release digestive enzymes (metalloproteinases) that sterilize and stimulate vascular growth. Additionally, their alkaline secretions have broad-spectrum antimicrobial activity against methicillin-resistant Staphylococcus aureus (MRSA), Pseudomonas, and the gram-positive aerobic bacteria commonly found in venous ulcers [9]. Through these mechanisms, they facilitate a transition away from the pro-inflammatory state to a pro-angiogenic phase with matrix remodeling and tissue healing [10].

Benefits of maggot therapy in wound healing are well described. In a case-control study by Armstrong et al. on patients with neuro-ischemic diabetic foot wounds and peripheral vascular disease, he demonstrated that maggot-treated patients required fewer days of antibiotics and healed their wounds at an average of four weeks faster than control patients [11]. Similarly, a cohort study carried out by Ronald Sherman showed that 85% of maggot-treated wounds were completely debrided compared to 48% in the group treated by conventional therapy alone. Within three weeks, maggot-treated wounds contained one-third of the necrotic tissue (p=0.05) and twice the granulation tissue (p<0.001), compared to controls [12].

The benefits of maggot therapy in venous ulcers have also been described by Wayman et al. [13]. In his study, he compared larval debridement therapy (LDT) to standard therapy with hydrogel for the treatment of necrotic venous ulcers. His study showed effective debridement after one application of LDT in 6/6 patients vs 4/6 patients in the hydrogel or control group who still required hydrogel dressings after one month of application. The LDT group was also shown to be more cost-effective than the control group (p<0.05). Despite strong evidence supporting the effectiveness of maggot debridement, it is still not widely accepted or used clinically. This may be due to several factors.

First, standardized research in the form of randomized controlled trials explaining how and when to utilize maggots is rare. Additional studies are needed to develop knowledge in this area and establish guidelines for use. Second, there is known psychological discomfort that accompanies the use of live organisms medically. Both these reasons, we feel, can be remedied. 

Recent technological advances have led to more aesthetically pleasing modes of maggot therapy administration. The most common are “maggot containment dressings” and “maggot confinement dressings”. The first is a layered net-like sheet that is placed over a patient’s wound and keeps maggots in place while allowing them to freely roam. The “confinement dressing” is a maggot containing pouch that is placed over a patient’s wound allowing restricted access. Both physician and patients need to be made aware of these modalities that have been shown to significantly reduce discomfort and are less conspicuous [4]. 

Maggot debridement therapy has been shown, however, to be contraindicated in open abdominal cavity wounds due to the risk of organ infestation. It is also contraindicated in dry wounds as maggots need a moist environment to survive, septic arthritis, patients on immunosuppressive therapy, and pyoderma gangrenosum [14]. It has also been postulated that extra caution is needed when administering maggot debridement therapy near large arteries or veins.

This case of a patient, who repeatedly failed conventional therapy but experienced markedly improved healing after an incidental maggot infestation, highlights the potential and underutilized benefit of maggot therapy in recalcitrant ulcers. Despite increased pain, his ulcer was markedly improved, showing decreased size and healthy granulation tissue.

To date, the debridement efficacy of maggot wound therapy is well supported by literature. Still, its practice is underutilized in hospital and clinical settings. This report aims to emphasize newer and more appealing delivery mediums for maggot therapy, hoping this will improve prescription and patient’s acceptance of the therapy. With conventional ulcer management strategies failing and antimicrobial resistance becoming increasingly common, maggot debridement therapy should be considered more frequently. 

## Conclusions

Maggot therapy is a well-supported practice that has fallen out of favor. This case, of a patient who failed conventional therapy but improved after a maggot infestation highlights the underutilized benefit of maggot therapy in recalcitrant ulcers, specifically venous stasis ulcers. Through continued research and education, we believe the biases noted by patients and practitioners that dissuade them from maggot therapy can be remedied. 
